# Phosphatidylcholine causes adipocyte-specific lipolysis and apoptosis in adipose and muscle tissues

**DOI:** 10.1371/journal.pone.0214760

**Published:** 2019-04-08

**Authors:** Tae Woo Jung, Taekwang Park, Jinwoo Park, Uiseok Kim, Hyun Dong Je, Hyeong-Dong Kim, Seong-Wan Cho, A. M. Abd El-Aty, Jin-Ho Song, Hyoung-Chun Kim, Yong Kyoo Shin, Ji Hoon Jeong

**Affiliations:** 1 Department of Pharmacology, College of Medicine, Chung-Ang University, Heuksuk-dong, Dongjak-gu, Seoul, Republic of Korea; 2 Department of Pharmacology, College of Pharmacy, Catholic University of Daegu, Gyeongsan, Republic of Korea; 3 Department of Physical Therapy, College of Health Science, Korea University, Seoul, Republic of Korea; 4 Department of Pharmaceutics & Biotechnology, Konyang University, Daejeon, Republic of Korea; 5 Department of Pharmacology, Faculty of Veterinary Medicine, Cairo University, Giza, Egypt; 6 Department of Medical Pharmacology, Medical Faculty, Ataturk University, Erzurum, Turkey; 7 Neuropsychopharmacology and Toxicology Program, College of Pharmacy, Kangwon National University, Chunchon, Republic of Korea; Tohoku University, JAPAN

## Abstract

Phosphatidylcholine (PPC) formula has been therapeutically used to reduce areas of localized fat. However, no single research has been carried out on its effect on a variety of cells in adipose and muscle tissues. Herein, the current study aimed to explore the activity of PPC on different cells in adipose and muscle tissues and to investigate the molecular mechanisms contributing to the effects of PPC on lipolysis and apoptosis. mRNA expression levels of various genes were measured by quantitative real-time PCR. Protein expression levels were observed through Western blotting and cell viability was measured by MTT assay. Lipolysis and caspase 3 activity assay were performed using commercial kits. PPC induces lipolysis and apoptosis in adipocytes (3T3-L1), but not in the other tested cells, including skeletal muscle cells (C2C12 myocytes), endothelial cells (HUVEC), and fibroblasts (BJ). The possible role of TNFα and IL-1β-mediated pathways on the effects of PPC was also revealed. We confirmed that treatment with PPC caused lipolysis and apoptosis in a dose-dependent manner (only in 3T3-L1 adipocytes). The effect of PPC observed in 3T3-L1 adipocytes was not evident in C2C12 myocytes, HUVEC, and fibroblasts. PPC also increased TNFα and IL-1β expression and release in 3T3-L1 adipocytes in a dose-dependent fashion, but not in C2C12 myocytes, HUVEC, and BJ. Suppression of TNFα or IL-1β reversed PPC-induced lipolysis and apoptosis in 3T3-L1 adipocytes, suggesting that PPC could promote adipocyte-specific lipolysis and apoptosis through TNFα and IL-1β-mediated signaling. We conclude that the specific activity of PPC on adipocyte in adipose without other tissue damages can be an effective approach for melting lipid.

## Introduction

Mesotherapy is a non-surgical, minimally invasive technique of drug delivery into the mesoderm to treat local regions [1]. The major function of this system is to increase the dose of a drug and exhibits strong therapeutic effects on many infirmities, such as fat embolism, hyperlipidemia, local pain, and hepatic problems [2]. Phosphatidylcholine (PPC) is a lecithin-derived phospholipid naturally found in egg yolk, soybeans, and milk [3]. This component suppresses lipid accumulation and ameliorates hepatic disorders resulted from hepatic lipid accumulation, myocardial ischemia, and dementia [3–5]. Currently, PPC-based formula has been used for treatment of local lipid accumulation via regulation of fat lipolysis [6]. Moreover, the size of lipoma is reduced after intralesional injection of PPC [7].

Bile salts, such as sodium deoxycholate (SD), have been used to improve of the hydrophilicity of PPC [8] before being used in open label clinical trials [9]. Alternative to liposuction, PPC-based formulation has been used to reduce partial fat tissue as a non-surgical method [10]. Previously, several studies have demonstrated that subcutaneous injection of PPC-based formula could result in fat dissolution [11, 12]. However, owing to its surfactant characteristics, SD causes severe pain (through necrosis and inflammation) and stimulates fat degradation in a non-specific way [13]. In our previous study, we have reported the effect of PPC-based formulation without SD together with its lipolytic activity in 3T3-L1 adipocytes via TNFα-mediated pathway [14]. It has to be noted that the selectivity of PPC-based formulation without SD to various cell types remains unclear, although it was revealed that a formula comprising PPC specifically affects adipocytes and has less effect on preadipocyte viability [15].

Taken together the present study was designed to elucidate the effects of a formula comprising PPC without SD on the expression of lipolytic cytokines, including tumor necrosis factor alpha (TNFα), interleukin 1 beta (IL-1β), and interferon gamma (IFNγ), and apoptosis in various cell types (adipocytes, myocytes, vascular endothelium, and fibroblast cells). We further explored the role of TNFα and IL-1β in PPC-mediated lipolysis and apoptosis in adipocytes.

## Materials and methods

### Cell cultures and reagents

3T3-L1 fibroblast cells (ATCC, Manassas, VA, USA) were cultured in Dulbecco`s modified eagle medium (DMEM) (Invitrogen, Carlsbad, CA, USA) supplemented with 10% fetal bovine serum (FBS; Invitrogen), 100 U/mL penicillin, and 100 μg/mL streptomycin (Invitrogen) [14, 16]. For differentiation, cells were grown to confluence for 3–5 days without changing the medium. At this point (considered as day zero), 3T3-L1 preadipocytes were differentiated by incubation in culture medium containing 2 μg/mL insulin, 400 μM methyl-isobutyl-xanthine, and 200 nM dexamethasone. Cells were cultured 72 h later with culture medium supplemented with 1 μg/mL insulin, and then every 48 h with fresh culture medium. 3T3-L1 adipocytes are kept in culture medium containing insulin until day 6 and then fed with normal medium without insulin every 48 h. Experiments were performed on day 10 and 13 of differentiation (S1 Fig). The mouse skeletal muscle cell line C2C12 (ATCC) was cultured in DMEM (Invitrogen) supplemented with 10% FBS (Invitrogen), 100 U/mL penicillin, and 100 μg/mL streptomycin (Invitrogen). C2C12 were boosted with 2% horse serum for myocyte differentiation. Human umbilical vein endothelial cells (HUVEC; ATCC) were cultured on 0.2% gelatin-coated culture plates in M200PRF medium (Invitrogen) with a low serum growth supplement (Invitrogen). Normal foreskin fibroblast cells (BJ) were grown in ATCC-formulated Eagle's Minimum Essential Medium (ATCC) supplemented with 10% FBS (Invitrogen), 100 U/mL penicillin, and 100 μg/mL streptomycin (Invitrogen). Cells were cultured in a humidified atmosphere of 5% CO_2_ at 37°C. PPC (LIPOID S-100, Lipoid GmbH, Ludwigshafen, Germany) was conjugated to 0.8% bovine serum albumin (BSA, fatty acid free grade; Sigma, St. Louis, MO, USA) dissolved in serum free culture media [14]. Lipoid S-100 (> 94% purity), a highly purified PPC from soybean, is clearly dissolved in ethanol at 20°C. In all experiments, cells were treated with PPC-BSA complex for 24 h and vehicle-0.8% BSA was set as a control.

### Western blotting analysis and antibodies

Proteins from 3T3-L1 adipocytes were extracted by lysis buffer (PRO-PREP; Intron Biotechnology, Seoul, Republic of Korea) for 90 min at 4°C. Protein samples (30 μg) were loaded to 10% SDS-PAGE, transferred to a nitrocellulose membrane (Amersham Bioscience, Westborough, MA, USA), and probed with primary antibody followed by secondary antibody conjugated with horse radish peroxidase (Santa Cruz Biotechnology). The samples were detected with enhanced chemoluminescence (ECL) kits [14, 16]. Anti-TNFα (1:1000), anti-IL-1β (1:1000), anti-NFκB (1:1000), and anti-β-actin (1:3000) (Santa Cruz Biotechnology, Santa Cruz, CA, USA) were used.

### Total RNA extraction and quantitative real-time PCR

Total RNAs from 3T3-L1 adipocytes were extracted using TRIzol reagent (Invitrogen). RNAs were transcribed to cDNA at 40°C for 60 min in a 20-μL cocktail containing 5× reverse transcriptase (RT) buffer, 15 mM dNTPs (300 U), Maloney murine leukemia virus reverse transcriptase (MMLV-RT) (Promega, Madison, WI, USA) and 100 pmol oligo-dT primer. cDNA concentration was estimated by the quantitative RT-PCR method using 2× iQTM SYBR Green Supermix (Bio-Rad, Hercules, CA, USA), to measure the mRNA levels of each gene. Amplification was performed using a CFX Connect Real-Time PCR Detection System (Bio-Rad) under the following conditions: 94°C for 5 min, 40 cycles at 94°C for 20 s, and 58°C for 35 s. Melting-curve analysis was used to verify the specificity of the PCR products. The comparative threshold method was used to calculate the relative amounts of mRNA in the experimental samples compared to the controls. Gene expression was normalized to the expression levels of β-actin. The relative fold-change of each gene was calculated using the method of delta-delta cycle threshold (ddCT). The following PCR conditions were used: 94°C for 5 min, 94°C for 10 s and 60°C for 2 min for 40 cycles. Primers were designed using Primer Express. Mouse TNFα (Mm00443258_m1), IL-1β (Mm00434228_m1), and β-actin (Mm02619580_g1) primers were purchased from Applied Biosystems (Lincoln Centre Drive Foster City, CA, USA)

### MTT assay

3-(4, 5-dimethylthiazolyl-2)-2, 5-diphenyltetrazolium bromide (MTT) was dissolved in phosphate buffered saline (PBS; Invitrogen) to prepare a working solution of 2 mg/mL; 100 μL MTT was added to each well. Cells were incubated at 37°C, 5% CO_2_, 95% air, and 100% humidity. After 3 h, the MTT solution was replaced with 100 μL of DMSO. For thorough mixing, the plates were incubated at room temperature for 10 min, and the optical density (OD) was measured using a multi-plate reader at a test wavelength of 570 nm and a reference wavelength of 630 nm.

### Caspase 3 activity assay

Caspase 3 activity assay was performed according to the manufacturer`s protocol using Caspase 3 Assay Kit (Colorimetric) (Abcam, San Jose, CA, USA).

### Enzyme linked immunosorbent assay (ELISA)

Cell secreted TNFα (Cat. No.: MTA00B), IL-1β (Cat. No.: DY401-05), and IFNγ (Cat. No.: MIF00) levels were measured using the corresponding ELISA kits (R&D Systems, Minneapolis, MN, USA) following the manufacturer’s instructions.

### Gene silencing using siRNAs

Small interfering (si) RNA oligonucleotides (0–20 nmol/L) targeting TNFα, IL-1β, and NFκB were purchased from Santa Cruz Biotechnology and transfected to suppress gene expression. Transfection using lipofectamine 3000 (Invitrogen) was performed according to the manufacturer`s directions

### Lipolysis assay

Lipolysis using colorimetric assay was performed using Abcam lipolysis assay kit following the manufacturer`s guidelines.

### Statistical analysis

All statistical analyses were performed using SPSS/PC statistical program (version 13.0 for Windows; SPSS, Chicago, IL, USA). Results are presented as the fold of the highest values (means ± SEM). All experiments were conducted in triplicates. Student`s *t* test or one-way ANOVA were used for statistical analysis.

## Results

### The effects of PPC on apoptosis in various cell types

**Fig 1:**
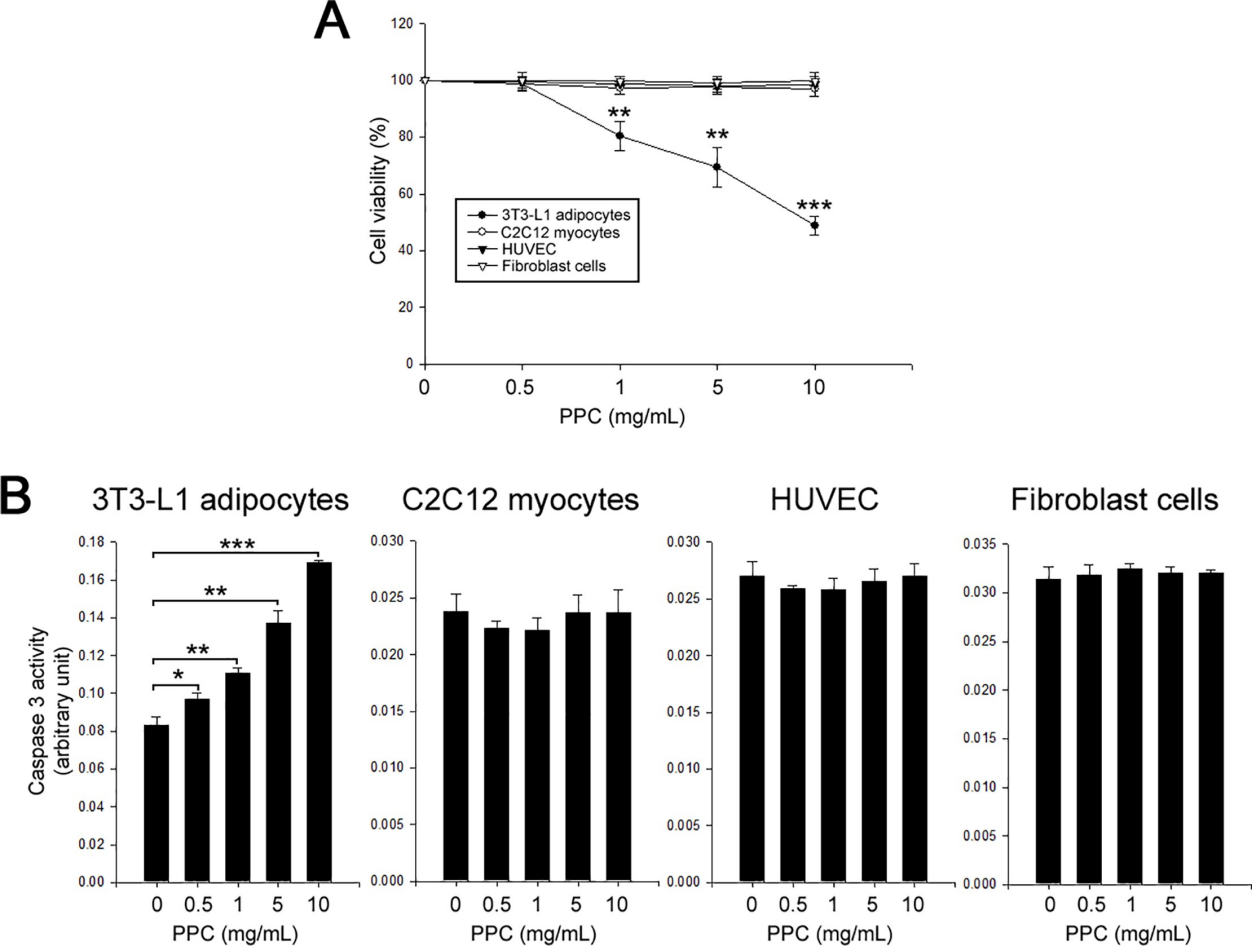
PPC induces apoptosis in adipocytes, but not in non-adipocytes. 3T3-L1 adipocytes, C2C12 myocytes, HUVEC, and fibroblast cells were treated with different concentrations (0–10 mg/mL) of PPC for 24 h. Cells were assessed by MTT assay to determine viability (A) and caspase 3 activity (B). Means ± SEM were calculated from three independent experiments. *** *P* < 0.001, **P < 0.01, and *P < 0.05 compared to culture control.

To evaluate the effects of PPC on apoptosis in various cell types, we treated 3T3-L1 adipocytes, C2C12 myocytes, HUVEC, and fibroblasts with PPC (0–10 mg/mL, 24 h). Cell viability was measured using MTT assay. PPC-based formula decreased 3T3-L1 cell viability and augmented caspase 3 activities in a dose-dependent manner. Interestingly, PPC treatment did not affect both cell viability and caspase 3 activities in C2C12 myocytes, HUVEC, and fibroblasts (Fig 1).

### The role of TNFα and IL-1β in PPC-induced lipolysis and apoptosis in 3T3-L1 adipocytes

**Fig 2:**
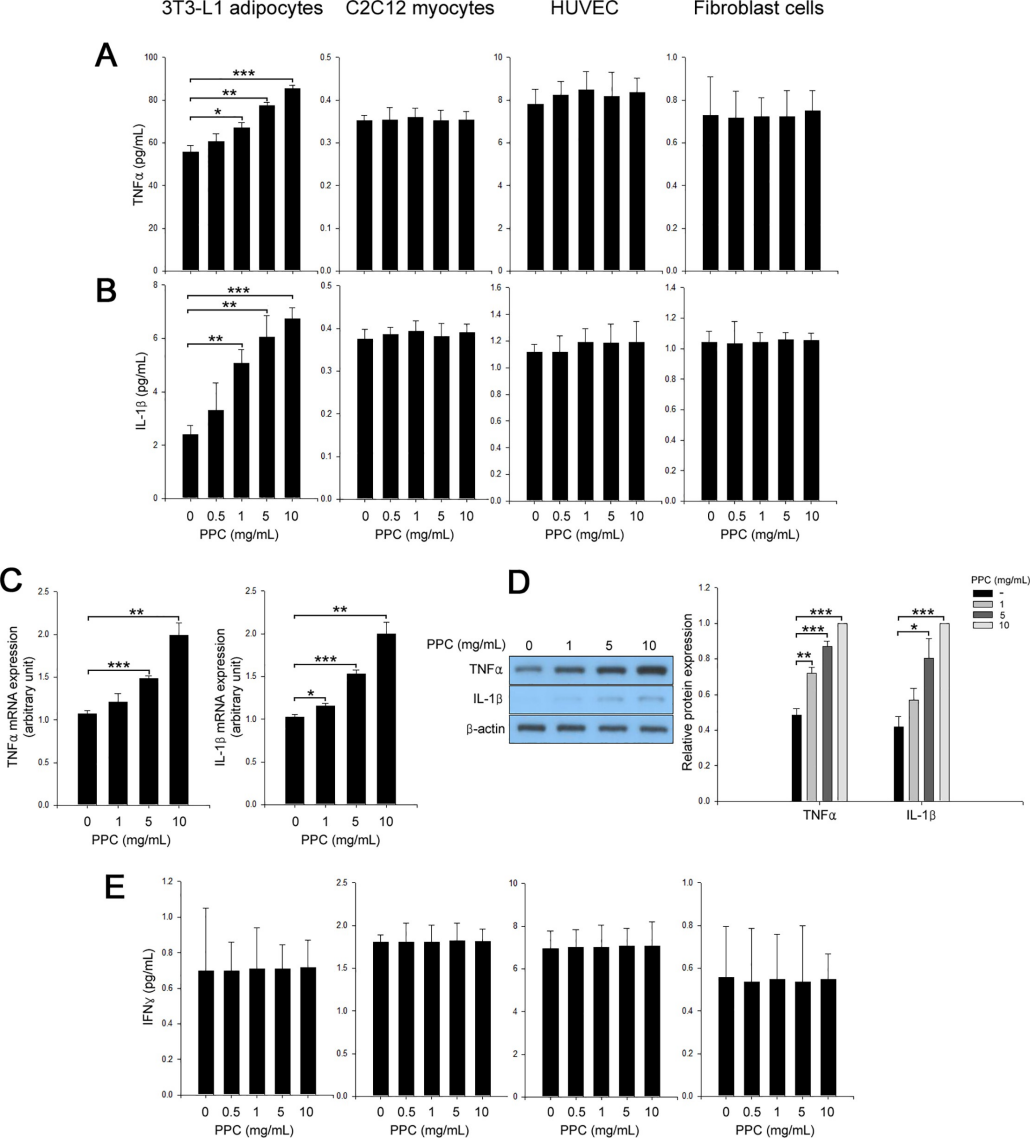
PPC specifically induces TNFα and IL-1β secretion and expression in adipocytes, but not in non-adipocytes. 3T3-L1 adipocytes, C2C12 myocytes, HUVEC, and fibroblast cells were treated with different concentrations (0–10 mg/mL) of PPC for 24 h. Culture media were used to measure TNFα (A) and IL-1β (B) secretion. TNFα and IL-1β mRNA expression was determined by quantitative real-time PCR analysis (C). Cell extracts were analyzed by Western blotting to determine TNFα and IL-1β expression (D). Culture media were used to measure IFNγ (E) secretion. Means ± SEM were calculated from three independent experiments. *** *P* < 0.001, **P < 0.01, and *P < 0.05 compared to culture control.

**Fig 3:**
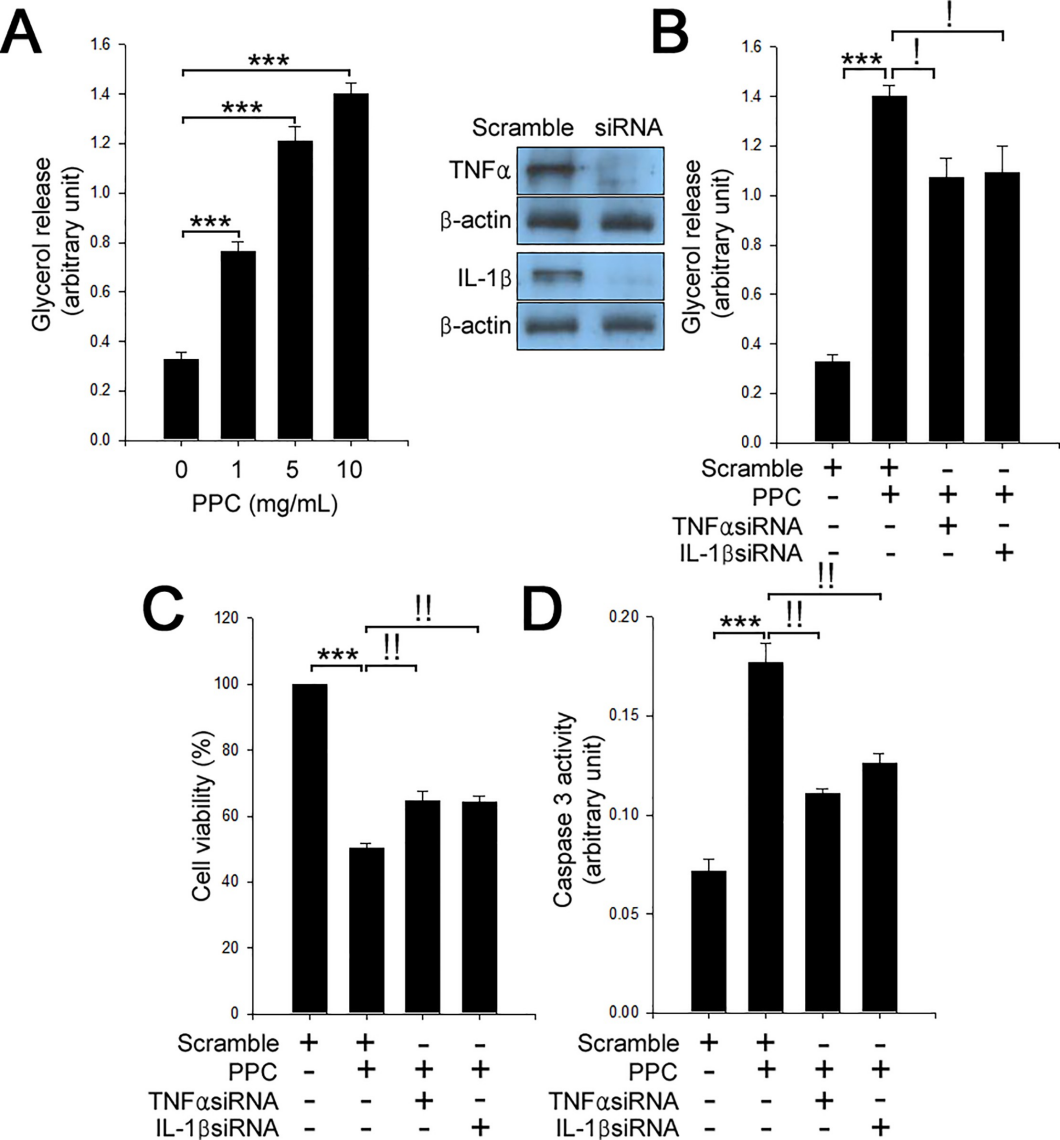
TNFα and IL-1β contribute to PPC-mediated lipolysis and apoptosis in adipocytes. (A) 3T3-L1 adipocytes were treated with different concentrations (0–10 mg/mL) of PPC for 24 h. Cells were assessed by lipolysis assay. Scramble and TNFα or IL-1β siRNAs-transfected 3T3-L1 adipocytes were treated with PPC (10 mg/mL) for 24 h. Cell extracts were measured by lipolysis assay (B), MTT assay to determine cell viability (C), and caspase 3 activity (D). Means ± SEM were calculated from three independent experiments. ***P < 0.001 compared to control 3T3-L1 adipocytes. ^!^*P* < 0.001 and ^!!^*P* < 0.01 compared to the levels in 3T3-L1 adipocytes treated with PPC.

The three cytokines; TNFα [17, 18], IL-1β [19, 20], and IFNγ [21, 22] are known to contribute to lipolysis and apoptosis. Thus, we examined the expression of these cytokines in various cell types. There were no changes of secretion of these cytokines in C2C12 myocytes, HUVEC, and fibroblasts. However, we found that treatment of 3T3-L1 adipocytes with PPC augmented the expression and secretion of TNFα and IL-1β in a dose-dependent manner (Fig 2A–2D). PPC-based formula did not influence the expression and secretion levels of IFNγ mRNA (Fig 2E). In line with our previous results [14], treatment of 3T3-L1 adipocytes with PPC stimulated lipolysis in a dose-dependent manner (Fig 3A). Subsequently, we investigated the role of TNFα and IL-1β in PPC-induced lipolysis and apoptosis. Suppression of TNFα [14] or IL-1β by each siRNA suppressed the PPC-induced lipolysis and apoptosis in 3T3-L1 adipocytes (Fig 3B–3D).

### PPC-mediated induction of TNFα and IL-1β expression via NFκB-dependent pathway

**Fig 4:**
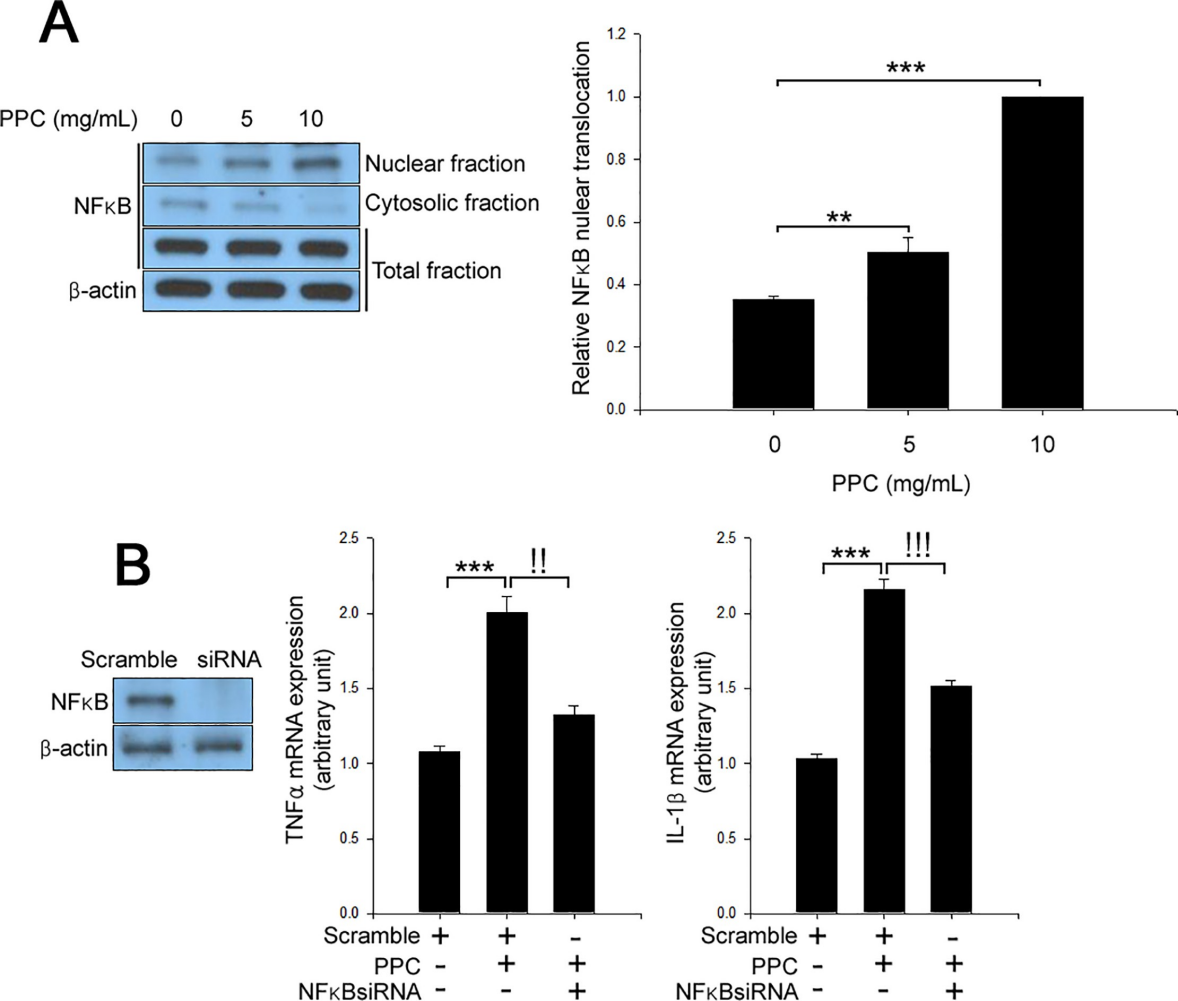
PPC regulates TNFα and IL-1β expression through NFκB nuclear translocation. (A) 3T3-L1 adipocytes were treated with different concentrations (0–10 mg/mL) of PPC for 24 h. Cell extracts were analyzed by Western blot. (B) Scramble and NFκB siRNAs-transfected 3T3-L1 adipocytes were treated with PPC (10 mg/mL) for 24 h. Cell extracts were analyzed by Western blotting. Means ± SEM were calculated from three independent experiments. ***P < 0.001 and **P < 0.01 compared to control level in 3T3-L1 adipocytes. ^!!!^*P* < 0.001 and ^!!^*P* < 0.01 compared to the levels in 3T3-L1 adipocytes treated with PPC.

Since TNFα [23] and IL-1β [24] promoter regions contain NFκB binding sites, we next examined the role of NFκB in PPC-induced TNFα and IL-1β expression. Nuclear translocation of NFκB was increased in 3T3-L1 adipocytes treated with PPC in a dose-dependent manner (Fig 4A). siRNA targeting NFκB mitigated the effects of PPC on TNFα and IL-1β mRNA expression in 3T3-L1 adipocytes (Fig 4B). Furthermore, suppression of NFκB by siRNA abrogated the effects of PPC on lipolysis and apoptosis (S2 Fig).

## Discussion

Non-surgical treatment for localized lipid accumulation via chemical lipolysis has received much attention [25], owing to its economic efficiency [26]. Injection of various PPC-based formula has been used for treatment of localized fat deposits [9]. A PPC formulation incorporate both PPC and SD [9]. SD serves as a detergent and shows a strong lipolytic activity [27]. The most common adverse effects, including swelling, bruising, and sensitizing treated skin have been reported for PPC-based formulation [6]. As SD causes severe inflammation and necrosis in human lipoma [27], it is thus believed that the side effect profiles of PPC formulations are attributed to SD not to PPC itself. It has been proposed that SD causes necrosis and inflammation in a non-specific manner [28–31]. It was also reported that PPC alone without SD could induce lipolysis and apoptosis in adipocytes [14, 32]. In the present study, MTT and caspase 3 activity assays indicated that PPC induced apoptosis in 3T3-L1 adipocytes, but not in C2C12 myocytes, HUVEC, and fibroblast cells. In line to previous reports [14, 25, 32], our current results are limited; PPC shows the possibility of destroying only adipose tissue without damaging other tissues. However, further *in vivo* studies are necessitated to reflect the effects of PPC on the viability of other non-adipocytes.

TNFα, a cytokine produced primarily from mature adipocytes and macrophages, plays a pivotal role in glucose and lipid metabolism [33, 34]. Treatment of TNFα increases lipolytic activity in adipocytes [33] and induces apoptosis in human adipocytes, rat brown adipocytes, and mouse white adipocytes [18, 35, 36]. Moreover, Zhao et al. have reported that TNFα could contribute to apoptosis in 3T3-L1 preadipocytes and mouse preadipocytes through SOC3-dependent signaling [37]. Recently, Jung et al. have also found that PPC could promote adipocyte lipolysis and apoptosis through TNFα-mediated pathway [14]. On the other hand, IL-1β, a proinflammatory cytokine produced by monocytes, macrophages [38], and adipocytes [39], suppresses expression of adipogenesis-associated genes and stimulates lipolysis in human primary adipocytes [19]. Furthermore, IL-1β modulated by TNFα in mouse adipocytes has been reported to cause hepatic insulin resistance [40]. Here, we demonstrated that PPC augmented TNFα [14] and IL-1β expression and release in 3T3-L1 adipocytes, but not in C2C12 myocytes, HUVEC, and fibroblast cells. We previously reported that NFκB participates in PPC-induced TNFα expression [14] and increased IL-1β expression through NFκB-mediated pathway. Thence, suppression of TNFα and IL-1β expression attenuated PPC-mediated lipolysis and apoptosis in 3T3-L1 adipocytes. These results suggest that NFκB-regulated TNFα and IL-1β, play a pivotal role in PPC-mediated lipolysis and apoptosis in adipocytes. To explore other mechanisms of lipolysis and apoptosis in 3T3-L1 adipocytes after PPC treatment, we further examined the effect of PPC on mRNA expression of IFNγ that was involved in lipolysis in adipocytes [22] and apoptosis [41]. Unexpectedly, PPC did not affect IFNγ mRNA expression, showing that PPC promotes lipolysis and induces apoptosis independent of IFNγ. Further studies for TNFα and IL-1β in null mice are required to verify the mechanism of action of PPC mediated through the TNFα and IL-1β-pathways. Moreover, non-adipose tissue of experimental mice should be injected to determine whether inflammation and apoptosis could occur following PPC injection. To clarify the reason why PPC selectively acts on adipocytes, it is important to find specific PPC receptors that exist only in adipocytes. Therefore, we are going to conduct a protein-protein interaction assay and receptor targeted DNA micro array.

However, Palumbo et al. have reported that PPC ameliorated SD-induced lipolysis in human primary adipocytes [42]. This discrepancy may be due to experimental conditions, such as culture condition, adipocyte differentiation levels, and species specificity. Further studies should be undertaken to interpretate these different results.

## Conclusions

**Fig 5:**
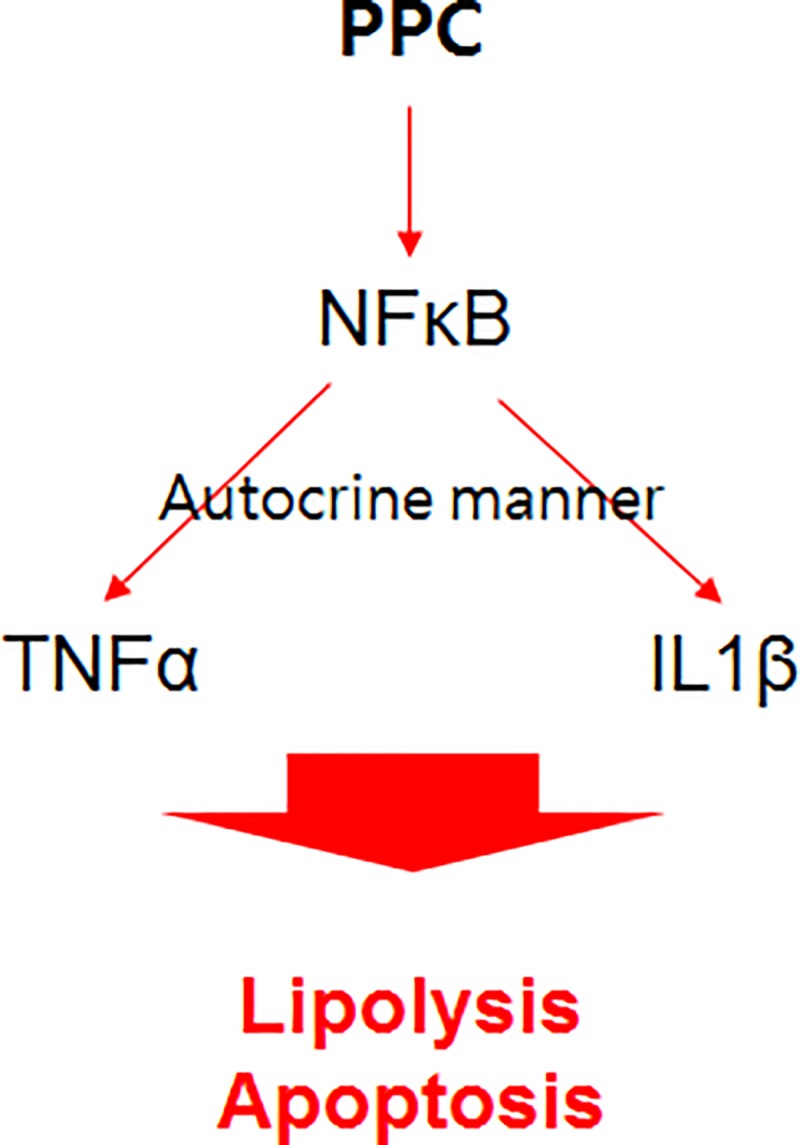
Schematic diagram for the effect of PPC on lipolysis and apoptosis in 3T3-L1 adipocytes.

PPC augmented TNFα and IL-1β expression, leading to lipolysis and apoptosis in 3T3-L1 adipocytes (Fig 5), but not in non-adipocytes (skeletal muscle, endothelial, and fibroblast cells). The current findings suggest that PPC alone can promote adipose tissue-specific reduction via the TNFα and IL-1β pathways without damage to other tissues. Based on the current results, we could propose the development of various substances that can selectively destroy adipose tissue either at the level of adipocyte-specific receptors or intracellular proteins for treatment of local obesity.

## Supporting information

S1 Fig Differentiation of 3T3-L1 cells. (TIF)

S2 Fig PPC promotes lipolysis and apoptosis via NFκB-mediated signaling in adipocytes. Scramble and NFκB siRNA-transfected 3T3-L1 adipocytes were treated with PPC (10 mg/mL) for 24 h. Cell extracts were measured by lipolysis assay (A), MTT assay to determine cell viability (B), and caspase 3 activity (C). Means ± SEM were calculated from three independent experiments. ***P < 0.001 and **P < 0.01 compared to control 3T3-L1 adipocytes. ^!!^*P* < 0.01 compared to the levels in 3T3-L1 adipocytes treated with PPC.
(TIF)

## Abbreviations

PPC: Phosphatidylcholine
siRNA: Small interfering RNA
TNFα: Tumor necrosis factor alpha
IL-1β: Interleukin 1 beta
