# Supplementary figures and images for: Phosphatidylcholine causes adipocyte-specific lipolysis and apoptosis in adipose and muscle tissues
Source: PLoS One. 2019 Apr 8;14(4):e0214760. doi: 10.1371/journal.pone.0214760 (PMC6453443; doi:10.1371/journal.pone.0214760)

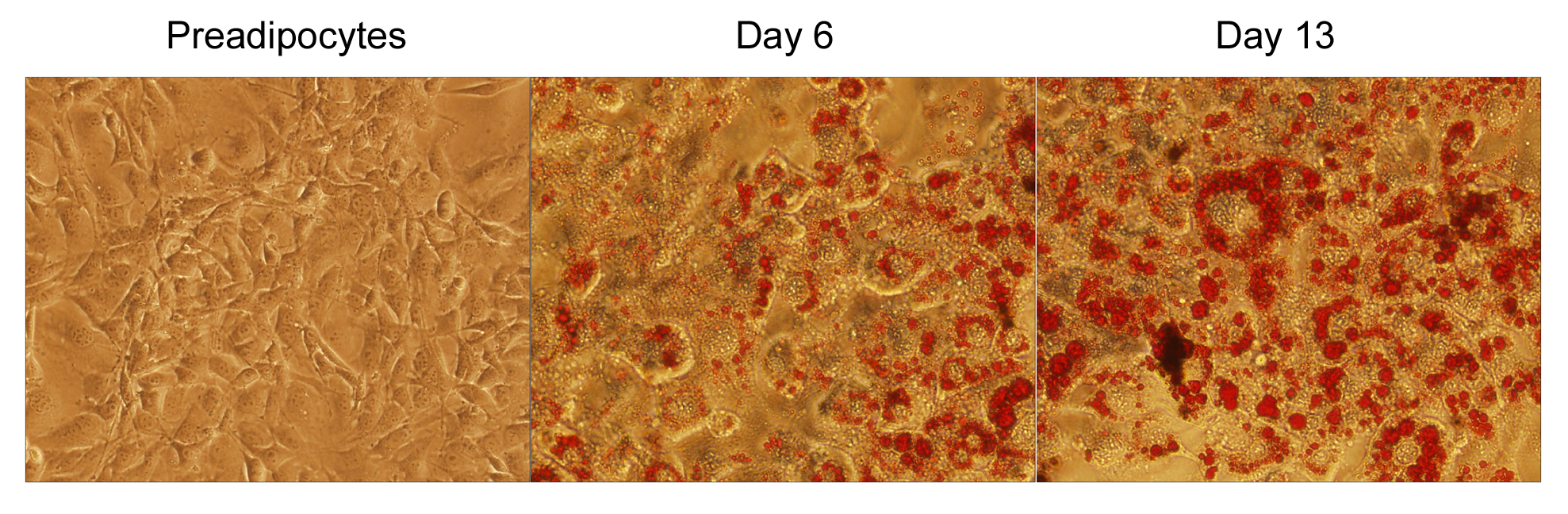

Supplement: S1 Fig — (TIF) [file pone.0214760.s001.tif]

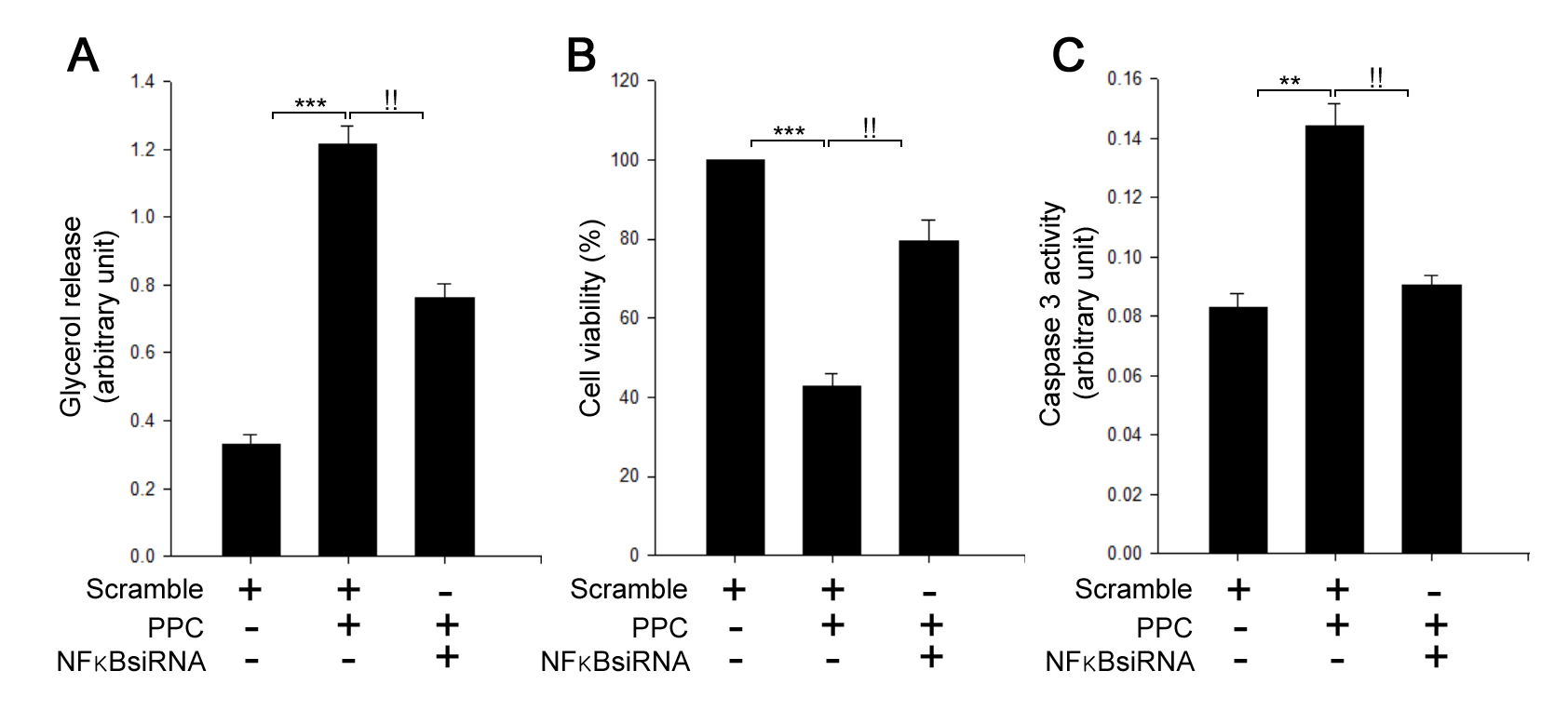

Supplement: S2 Fig — Scramble and NFκB siRNA-transfected 3T3-L1 adipocytes were treated with PPC (10 mg/mL) for 24 h. Cell extracts were measured by lipolysis assay (A), MTT assay to determine cell viability (B), and caspase 3 activity (C). Means ± SEM were calculated from three independent experiments. ***P < 0.001 and **P < 0.01 compared to control 3T3-L1 adipocytes. !!P < 0.01 compared to the levels in 3T3-L1 adipocytes treated with PPC. (TIF) [file pone.0214760.s002.tif]
